# Dietary Exposure to 2,2′,4,4′-Tetrabromodiphenyl Ether (PBDE-47) Alters Thyroid Status and Thyroid Hormone–Regulated Gene Transcription in the Pituitary and Brain

**DOI:** 10.1289/ehp.11570

**Published:** 2008-08-01

**Authors:** Sean C. Lema, Jon T. Dickey, Irvin R. Schultz, Penny Swanson

**Affiliations:** 1 Physiology Program, Northwest Fisheries Science Center, National Oceanic and Atmospheric Administration, Seattle, Washington, USA; 2 School of Aquatic and Fishery Sciences, University of Washington, Seattle, Washington, USA; 3 Marine Sciences Laboratory, Battelle, Pacific Northwest National Laboratory, Sequim, Washington, USA

**Keywords:** basic transcription element-binding protein, brain, endocrine disruption, PBDE-47, polybrominated diphenyl ethers, thyroid hormone, thyroid hormone receptor, thyroid-stimulating hormone, thyrotropin

## Abstract

**Background:**

Polybrominated diphenyl ether (PBDE) flame retardants have been implicated as disruptors of the hypothalamic-pituitary-thyroid axis. Animals exposed to PBDEs may show reduced plasma thyroid hormone (TH), but it is not known whether PBDEs impact TH-regulated pathways in target tissues.

**Objective:**

We examined the effects of dietary exposure to 2,2′,4,4′-tetrabromodiphenyl ether (PBDE-47)—commonly the highest concentrated PBDE in human tissues—on plasma TH levels and on gene transcripts for glycoprotein hormone α-subunit (GPHα) and thyrotropin β-subunit (TSHβ) in the pituitary gland, the autoinduced TH receptors α and β in the brain and liver, and the TH-responsive transcription factor basic transcription element-binding protein (BTEB) in the brain.

**Methods:**

Breeding pairs of adult fathead minnows (*Pimephales promelas*) were given dietary PBDE-47 at two doses (2.4 μg/pair/day or 12.3 μg/pair/day) for 21 days.

**Results:**

Minnows exposed to PBDE-47 had depressed plasma thyroxine (T_4_), but not 3,5,3′-triiodothyronine (T_3_). This decline in T_4_ was accompanied by elevated mRNA levels for TStHβ (low dose only) in the pituitary. PBDE-47 intake elevated transcript for TH receptor αin the brain of females and decreased mRNA for TH receptor β in the brain of both sexes, without altering these transcripts in the liver. In males, PBDE-47 exposure also reduced brain transcripts for BTEB.

**Conclusions:**

Our results indicate that dietary exposure to PBDE-47 alters TH signaling at multiple levels of the hypothalamic-pituitary-thyroid axis and provide evidence that TH-responsive pathways in the brain may be particularly sensitive to disruption by PBDE flame retardants.

Polybrominated diphenyl ethers (PBDEs) are added to plastics, polyurethane foam, paints, and synthetic fabrics as a flame retardant. Recently, concerns have arisen about possible health impacts of PBDE exposure because studies have revealed rising PBDE levels in the tissues of humans and wildlife (Hites 2004; Law et al. 2003). The chemical structure of PBDEs resembles that of polychlorinated biphenyls (PCBs), and PBDEs may act similarly as disruptors of the hypothalamic–pituitary–thyroid axis (Birnbaum and Staskal 2004; Boas et al. 2006; McDonald 2002). In studies with rats, mice, and fish, *in vivo* PBDE exposure reduced plasma levels of the thyroid hormone (TH) thyroxine (T_4_) (Hallgren et al. 2001; Tomy et al. 2004; Zhou et al. 2002). Weanling rats given the commercial PBDE mixtures DE-71 and DE-79 at doses of 100–300 mg/kg/day showed up to 80% reductions in plasma T_4_ (Zhou et al. 2001), and American kestrels (*Falco sparverius*) administered PBDEs *in ovo* showed reduced T_4_ proportional to their tissue burdens of PBDE-47 and PBDE-99 (Fernie et al. 2005).

PBDEs are structurally similar to THs, and it has been hypothesized that PBDEs might interfere with TH transport and metabolism (Birnbaum and Staskal 2004; McDonald 2002). Supporting this idea, rats with reduced T_4_ after exposure to the PBDE mixture Bromkal 70-5DE also showed elevated hepatic uridine-5-diphospho-glucuronosyltransferase (UDP-GT) activity, suggesting that the decline in T_4_ may result in part from increased bilary excretion of conjugated TH (Skarman et al. 2005). Alternatively, some hydroxylated PBDEs bind the TH transport protein transthyretin with sufficient affinity to displace T_4_ (Hamers et al. 2006; Meerts et al. 2000). The mechanism by which PBDEs depress circulating T_4_ remains unclear, and it is also not known whether PBDE exposure impacts TH-regulated pathways in the brain or peripheral tissues. PBDEs appear to have only weak binding affinity for TH receptors (TRs) (Marsh et al. 1998), yet Schriks and co-workers (2007) found recently that one T_3_-like hydroxylated PBDE, 4′-hydroxy-2,4,6-tribromodiphenyl ether, increases T_3_-induced *TR* α activation in reporter-gene assays, whereas PBDE 209 inhibits T_3_ activation of both *TR*α and *TR*β. These new findings suggest that PBDE exposure may affect TH-regulated pathways in target tissues.

In the vertebrate brain, THs play key roles in regulating neural development and functioning (Koibuchi and Chin 2000; König and Neto 2002). THs influence neurogenesis by mediating the proliferation of neural progenitor cells, influencing dendritic and synapse formation, and regulating myelination (Gould and Butcher 1989; Porterfield and Hendrich 1993). In rodents, neurogenesis in the brain is induced by T_3_ (Uchida et al. 2005), and THs stimulate stem cell proliferation and neuronal differentiation in the olfactory system of mammals and fish (Lema and Nevitt 2004; Paternostro and Meisami 1994). Nevertheless, it remains unclear whether exposure to PBDEs impacts TH-mediated neural development (Porterfield 2000).

In the present study, we used teleost fish as an animal model for investigating the influence of PBDEs on neurogenesis and brain development. Teleost fish show cell proliferation and neural differentiation in the brain throughout adult life (Adolf et al. 2006; Lema et al. 2005; Zupanc et al. 2005), and the extent of neurogenesis in the adult fish brain greatly exceeds that in mammals (Cayre et al. 2002). Embryonic exposure of zebrafish (*Danio rerio*) to PBDE-47 has been found to lead to cardiac and morphologic defects that appear to be caused by a primary effect on neural function (Lema et al. 2007). Whether such neural and behavioral impacts of PBDE exposure result from disruption of TH signaling during brain development, however, has yet to be examined.

We tested the hypothesis that dietary exposure to PBDEs affects TH-regulated gene transcripts in target tissues in the adult fathead minnow (*Pimephales promelas*). The fathead minnow is a teleost model for assessing the toxic and endocrine-disrupting effects of chemical pollutants (Ankley and Villeneuve 2006), and oral exposure to the PBDE congener 2,2′,4,4′-tetrabromodiphenyl ether (PBDE-47) impaired reproductive activity in this species (Muirhead et al. 2006). We exposed adult minnows to a dietary source of PBDE-47, typically the most concentrated PBDE congener in humans and wildlife (Hites 2004; Schecter et al. 2005). We then examined the effects of PBDE-47 exposure on plasma T_4_ and T_3_ status and mRNA levels for thyrotropin β-subunit (TSHβ) and glycoprotein hormone α-subunit (GPHα) in the pituitary. We also examined how PBDE-47 affected key target tissues for THs by quantifying transcripts for the autoinduced *TR*α and *TR* β genes in the brain and liver, and by quantifying brain mRNA for basic transcription element-binding protein (BTEB), a TH-responsive transcription factor that regulates neural differentiation (Cayrou et al. 2002; Denver et al. 1999).

## Materials and Methods

### Animals and housing

Adult fathead minnows (*Pimephales promelas*) were obtained from Environmental Consulting & Testing (Superior, WI). Minnows were maintained at the Battelle Pacific Northwest Division in Sequim, Washington, under a 16 hr:8 hr light:dark photoperiod with water quality parameters of 24–26°C, 6.6–7.4 mg/L dissolved oxygen, and 8.1–8.3 pH for the duration of the experiment. All animals were treated humanely and with regard for alleviation of suffering, in accordance with guidelines of the Battelle Institutional Animal Care and Use Committee.

### Bioencapsulation of PBDE-47 in Artemia shrimp

We obtained PBDE-47 from ChemServices (> 99% purity; West Chester, PA). A stock solution of PBDE-47 was prepared by dissolving 10.0 mg/mL in hexane. One milliliter of stock solution was added to a 1-L Erlenmeyer flask, the hexane evaporated, and approximately 15,000 adult brine shrimp (*Artemia franciscana*) added and incubated overnight to bioencapsulate the PBDE-47 (Muirhead et al. 2006). Before dosing minnows with the bioencapsulated brine shrimp, aliquots of the *Artemia* were assayed for PBDE-47 concentration using gas chromatography.

### PBDE-47 exposures

We placed minnows in 38-L aquaria with one adult male and one adult female per aquarium. Each aquarium contained a 10.2-cm diameter clay pot that was split longitudinally to provide spawning substrate. Before beginning PBDE-47 exposure, the breeding pairs for all treatments (*n* = 9–11 pairs per treatment) were fed clean, frozen *Artemia* (San Francisco Bay Brand, Newark, CA) diluted 1:1 with sterile filtered seawater (~ 0.5 g wet weight/mL) *ad libitum* twice daily for 7 days, during which time we checked the spawning substrate every morning to confirm that each pair was reproductively active.

Following this 7-day period, minnows were fed PBDE-47 bioencapsulated *Artemia* (1 mL) twice daily for 21 days. Minnow pairs were given PBDE-47 either as a low dose (2.38 ± 0.63 μg PBDE-47/pair/day) or a high dose (12.30 ± 3.61 μg PBDE-47/pair/day). We selected these doses based on previous PBDE exposures with this species (Muirhead et al. 2006). A third, control group of minnow pairs continued to be fed *Artemia* not bioencapsulated with PBDE-47. We monitored spawning activity daily for the duration of PBDE-47 exposure, as described above.

After 21 days of PBDE-47 exposure, minnows were euthanized with tricaine methanesulfonate (Argent Chemical, Redmond, WA), and body mass (grams) and fork length (millimeters) were measured. Plasma was collected, and the pituitary gland, brain, and liver were dissected and frozen rapidly in liquid nitrogen, although the liver was first weighed to determine liver somatic index (LSI). We also dissected one gonad and immersed it in Bouin’s fixative for histologic analysis. After removal of the digestive tract, the remaining carcass of each animal was frozen to quantify body burdens of PBDE-47. All tissues were stored at −80°C.

### T_4_ and T_3_ radioimmunoassays

Plasma concentrations of T_4_ and T_3_ were measured by radioimmunoassay as described previously (Dickhoff et al. 1982) using anti-L-T_4_ (1:4,000) or anti-L-T_3_ antiserum (1:10,000) (Accurate Chemical & Scientific Corp., Westbury, NY) and ^125^I-labeled T_4_ or T_3_ (Perkin-Elmer, Waltham, MA). The intra-assay coefficient of variation was 4.1% for the T_4_ assay and 5.4% for the T_3_ assay. All samples were run in single assays. Given the small body size of fathead minnows, only the larger male sex provided sufficient plasma to quantify both T_4_ and T_3_ from the same individual. For that reason, we assayed T_3_ in males only.

### Cloning of cDNA for BTEB

We first identified and sequenced the cDNA for BTEB from the brain of fathead minnow using primers designed for zebrafish BTEB [GenBank accession no. AI979399 (National Center for Biotechnology Information 2008)]. First strand cDNA was amplified in a 50-μL polymerase chain reaction (PCR) containing 2 μg of total RNA from the brain under the thermal profile: 94°C for 2 min, followed by 30 cycles of 94°C for 30 sec, 50°C for 30 sec, and 72°C for 90 sec, and ending with 72°C for 10 min. The cDNA was purified and sequenced to provide a 151-bp partial sequence, which was used to design primers [see Supplemental Material, Table 1 (available online at http://www.ehponline.org/members/2008/11570/suppl.pdf)] to obtain the full length BTEB sequence (SMART RACE cDNA Amplification Kit; BD Biosciences, Palo Alto, CA). The full-length cDNA sequence for fathead minnow BTEB is available online [GenBank accession no. EF432310 (National Center for Biotechnology Information 2008)].

### Real-time quantitative reverse-transcribed PCR assays

We extracted total RNA from the pituitary gland using the MiniPrep RNeasy Kit (Qiagen, Inc., Valencia, CA) and from the brain and liver using Tri-Reagent (Molecular Research Center, Cincinnati, OH). Extracted RNA was quantified (NanoDrop Technologies, Wilmington, DE) and diluted to 15 ng/μL. Total RNA was reverse-transcribed (RT) in 15-μL reactions containing 3.0 μL 5× buffer and 1.5 μL dithiothreitol (DTT; Invitrogen, Carlsbad, CA), 0.75 μL deoxyribonucleotide triphosphate (dNTP) and 0.255 μL random hexamer (Promega, Madison, WI), 0.3 μL RNase inhibitor (20 U/μL; Applied Biosystems, Inc., Foster City, CA), 0.1875 μL Superscript II reverse transcriptase (Invitrogen), 6.0375 μL ddH_2_O (nuclease-free water; Sigma, St. Louis, MO), and 3.0 μL of total RNA template (15 ng/μL) under a profile of 25°C for 10 min, 48°C for 60 min, and 95°C for 5 min.

Primers and probes for real-time quantitative RT-PCR assays were designed for *TSH*β (GenBank accession no. DQ677879) (Lema et al. 2008), *GPH*α (DQ256072), *TR* α (DQ074645), *TR* β (AY533142) and *BTEB* (EF432310) from fathead minnow using Primer Express software (ABI). All primers and probes were synthesized by Integrated DNA Technologies (Coralville, IA) [see Supplemental Material, Table 2 (available online at http://www.ehponline.org/members/2008/11570/suppl.pdf)].

Quantitative RT-PCR reactions (25 μL) contained 12.5 μL Master Mix (ABI Universal MasterMix Reagent), 0.5 μL forward primer, 0.5 μL reverse primer, 0.5 μL probe, 8.0 μL nuclease-free H_2_O, and 3.0 μL of reverse-transcribed cDNA template. Reactions were run on an ABI 7700 Sequence Detector under a profile of 50°C for 2 min, 95°C for 10 min, and then 40–45 cycles of 95°C for 15 sec and 60°C for 1 min. All samples for each gene were run on a single 96-well plate. For each gene, we tested for DNA contamination by analyzing a total RNA sample that was not reverse-transcribed, and each run included duplicate samples lacking cDNA template. We used serial dilutions of total RNA from the experiment as a standard curve. Standard curve samples were run in triplicate, but samples themselves were not duplicated. We also quantified expression for *18S* (Universal 18S; ABI) as a potential normalizing gene. In the pituitary gland and liver, 18S transcript expression was affected by PBDE-47 treatment, and total RNA yield from the pituitary was insufficient to screen additional housekeeper genes. Therefore, instead of normalizing the genes of interest to 18S, pituitary and liver transcripts were expressed relative to the total yield of RNA. Relative gene transcript expression was subsequently calculated using the serially diluted standard curve, normalized to either total RNA template or 18S transcript in that tissue, and expressed as a relative level by dividing the measured values by the mean of a designated control group.

### Gonad histology

Gonad samples were immersed in Bouin’s fixative (24 hr) and transferred to 70% ethanol before being embedded in paraffin, sectioned longitudinally at 5 μm, and stained with hematoxylin and eosin. Stages of spermatogenesis and oogenesis as described by Leino et al. (2005) were quantified by stereology in three sections from each gonad.

### Quantification of PBDE-47 in body tissues

We used gas chromatography with electron capture detection (GC-ECD) to determine PBDE-47 concentrations in the carcass tissues. Carcasses were homogenized in deionized water and spiked with 20 μL of a 240 μg/mL solution of PCB 103 (wt/vol in hexane; Sigma) as an internal standard. Hexane (1 mL) was added, and the homogenate was vortexed (30 sec) and centrifuged at 3,000 × *g* (5 min). The hexane layer was then transferred to a GC-ECD autosampler vial and diluted 1:10 with hexane. Extraction efficiency of PBDE-47 was 90–95% in blank carcasses fortified with PBDE-47 at concentrations encompassing observed levels in experimental fish. The hexane extracts were analyzed on a Hewlett-Packard 5890 GC (Agilent Technologies, Santa Clara, CA) equipped with a DB-5 30-m, 0.25 μM capillary column and operated in split injection mode with a split ratio of 8:1. Standard curves prepared for PBDE-47 ranged from 0 to 237.6 μg.

### Statistical analyses

We used two-factor analysis of variance (ANOVA) models to examine the effects of treatment and sex on plasma T_4_, mRNA levels for TSHβ and GPHα in the pituitary, TRα and TRβ in the brain and liver, and BTEB in the brain. Samples that exceeded three standard deviations were consider outliers and excluded from analysis. We used a one-factor ANOVA model to test for effects on plasma T_3_. When a significant effect of treatment was found, pairwise comparisons were made using Dunnett’s tests. We used chi-square tests to compare the distribution of gonadal stages between treatments. Bonferroni-corrected pairwise *t*-test comparisons between the control and each treatment were then made within staging classes to identify which stages were altered by PBDE exposure. To examine how PBDE-47 affected spawning frequency, we used an analysis of covariance model with treatment, baseline spawning frequency as a covariate, and treatment × baseline spawning frequency as factors.

## Results

### Plasma thyroid hormones

**Figure 1:**
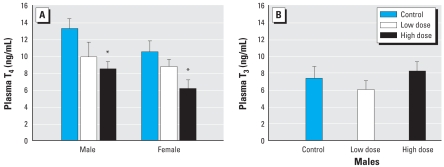
Exposure to PBDE-47 depressed circulating concentrations of total T_4_ in males and females (*A*), but had no effect on total T_3_ in males (*B*). *p < 0.05 compared with control.

We observed decreased plasma T_4_ levels in both sexes after dietary PBDE-47 exposure (*p* = 0.002; Figure 1). Males had higher plasma T_4_ levels than females (*p* = 0.0447), but this sex difference was independent of PBDE exposure. Plasma T_3_ levels in males were unaffected by PBDE-47.

### Pituitary gene transcripts

**Figure 2:**
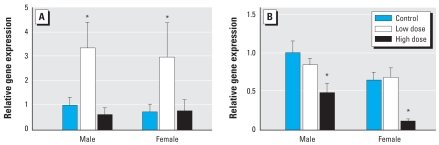
Dietary exposure to PBDE-47 altered relative transcripts levels for TSHβ (*A*) and GPHα (*B*) in the pituitary gland. Transcript levels are expressed relative to template RNA levels. *p < 0.05 compared with control.

At the lower exposure dose, PBDE-47 elevated gene transcripts for TSHβ in the pituitary gland (Figure 2; *p* = 0.0043). At the higher PBDE-47 dose, however, pituitary mRNAs for GPHα were reduced in both males and females (*p* < 0.0001) without a change in transcript for TSHβ.

### TR and BTEB mRNAs in the brain

**Figure 3:**
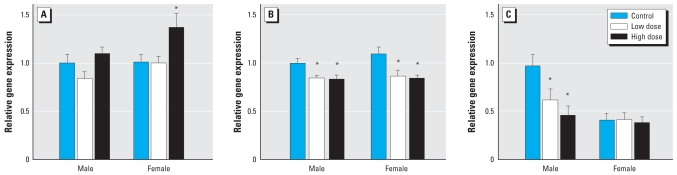
Dietary PBDE-47 exposure elevated mRNA levels for TRα in females (*A*), and reduced mRNA levels for TRβ in both sexes (*B*). PBDE-47 exposure also reduced gene transcripts for BTEB in the brain of male, but not female, minnows (*C*). Transcript levels are normalized to *18S*. *p < 0.05 compared to control.

Gene transcripts for *TR*α were elevated 37% in the brain of females (*p* = 0.002), but not males, exposed to the high PBDE-47 dose (Figure 3A). Transcript levels for *TR* α also differed between males and females (*p* = 0.0431). In both sexes, PBDE-47 exposure depressed brain TRβ mRNA levels at both PBDE dosing levels (Figure 3B; *p* = 0.001). There was no difference in brain TRβ transcript levels between sexes.

Dietary PBDE-47 exposure also altered mRNA abundance for the TH-regulated transcription factor BTEB, although this effect differed between sexes (Figure 3C; *p* = 0.029). In males, BTEB transcript was reduced in both the low and high PBDE-47 exposures. Females had lower levels of BTEB transcript than males (*p* = 0.0008), but expression in females was not affected by PBDE exposure.

### TR transcripts in the liver

LSI was greater in females than in males and was elevated 38% in males exposed to the high dose of PBDE-47 [*p* = 0.009; see Supplemental Material, Table 3 (available online at http://www.ehponline.org/members/2008/11570/suppl.pdf)]. Transcript levels for TRα and TRβ in the liver were not altered by PBDE-47 [see Supplemental Material, Figure 1 (available online at http://www.ehponline.org/members/2008/11570/suppl.pdf)]. Male minnows, however, had greater levels of TRβ mRNA in the liver than females (*p* < 0.0001). TRα mRNA levels did not vary between the sexes.

### Gonad staging and reproductive behavior

Male minnows exposed to PBDE-47 had fewer mature spermatozoa and more primary spermatocytes and spermatids compared with control males (low dose vs. control: χ^2^ = 17.78, *p* = 0.001; high dose vs. control: χ^2^ = 57.22, *p* < 0.001; see Supplemental Material, Table 4 (available online at http://www.ehponline.org/members/2008/11570/suppl.pdf)]. Although we observed fewer spermatozoa in males exposed to the high PBDE-47 dose, minnow pairs spawned at similar rates in all treatments.

### Tissue levels of PBDE-47

Minnows exposed to PBDE-47 had body burdens of PBDE-47 related to their dietary dose, but females had greater tissue concentrations of PBDE-47 than males. In the low-dose treatment, males had a body burden of 11.43 ± 1.24 μg PBDE-47/g carcass, whereas females had 20.07 ± 7.38 μg PBDE/g carcass. In the high-dose treatment, PBDE-47 levels were 64.62 ± 6.10 μg/g carcass in males and 107.60 ± 29.40 μg/g carcass in females. This sex difference in PBDE-47 body burdens corresponds to a previous study using similar dosing procedures with this species (Muirhead et al. 2006).

## Discussion

Dietary PBDE-47 depressed plasma T_4_ in male and female adult minnows. This reduction in T_4_ was associated with elevated transcript for TSHβ (low dose only) in the pituitary gland and changes in transcript expression for TH receptors at both exposure doses. The effects of PBDE-47 on TH receptor mRNA abundance were tissue specific. Transcripts for both TRα and TRβ in the liver were unaffected by PBDE-47. In the brain, however, PBDE exposure reduced levels of mRNA for TRβ in both sexes and elevated TRα mRNA in females. PBDE-47 also reduced brain mRNA levels of the TH-regulated transcription factor BTEB in males only.

The reduction in T_4_ observed in both sexes is consistent with previous studies showing that PBDE mixtures and single congeners can depress plasma T_4_ (Fernie et al. 2005; Hallgren et al. 2001). As was observed in the current study, these effects on T_4_ generally occur in the absence of any change in T_3_; although, if PBDE exposure occurs at greatly elevated levels, plasma levels of T_3_ may also be reduced (Zhou et al. 2001). The mechanism by which PBDEs depress T_4_ is not clear. Some researchers have hypothesized that the decreased T_4_ is caused by displacement of T_4_ from transport proteins (Hamers et al. 2006; Meerts et al. 2000), but quantitative structural-activity binding models indicate only weak affinity of PBDE-47 to transthyretin (TTR), although the hydroxylated form of PBDE-47, 6-OH-PBDE-47, has greater affinity (Harju et al. 2007). An experimental study using recombinant sea bream TTR likewise found that PBDE-47 has lower affinity for teleost TTR than either T_3_ or T_4_, whereas the affinity of 6-OH-PBDE-47 is greater than that of the endogenous hormones (Morgado et al. 2007). Hydroxylated PBDEs such as 6-OH-PBDE-47 are produced metabolically from parent compounds (Mörck et al. 2003), and metabolic conversion of PBDE-47 may have occurred in the minnows. TTR, however, is generally not the dominant TH transport protein in fish, and binding of PBDEs to thyroxin-binding protein and serum albumin has yet to be examined.

Our data show that the PBDE-induced reduction in peripheral T_4_ is accompanied by changes in pituitary mRNAs for TSH. TSH is composed of an α- and β-subunit with each subunit synthesized separately. The GPH α-subunit of TSH is identical to that of follicle-stimulating hormone (FSH) and luteinizing hormone (LH), so it is TSH’s β-subunit that determines the hormone’s functional specificity. In the present study, TSHβ mRNA was elevated in both sexes by the low PBDE-47 dose, and transcript for GPHα was depressed in both sexes at the high dose. The elevation in TSHβ mRNA at the lower dose is consistent with reduced negative feedback on the pituitary from the decline in circulating T_4_. At the higher PBDE dose, however, alternative regulatory mechanisms or toxic effects may occur. Supporting this idea, the testes of PBDE-47–exposed males in the high dose had fewer spermatozoa. A similar decline in spermatozoa was observed in fathead minnows given an oral dose of 28.7 μg PBDE-47/pair/day (Muirhead et al. 2006), suggesting that PBDE exposures at high doses may impact gametogenesis and pituitary feedback from gonadal steroids. Although the mechanism responsible for the PBDE-induced decline in circulating T_4_ cannot be discerned from alterations in pituitary mRNA levels alone, the reduced pituitary GPHα transcript may cause a reduction in bioactive TSH protein production and a decline in TH biosynthesis.

To test whether PBDE-47 exposure affects TH-mediated gene transcripts in target tissues, we quantified mRNAs for *TR* α and *TR*β in the brain and liver. TRs act as ligand-activated transcription factors by inducing or repressing the transcription of genes containing thyroid response elements (TREs). The genes for TRs themselves contain TREs, so that transcripts for TRα and TRβ are autoinduced by T_3_ (Lema SC, Dickey JT, Schultz IR, Swanson P, unpublished data; Liu et al. 2000). This autoinduction means that TR transcripts are markers for assessing TH-induced activation of gene transcripts in target tissues (Opitz et al. 2006). In fish and other vertebrates, distinct expression patterns of the TRα and TRβ isoforms suggest that TRs have tissue-specific and developmental state-specific functions (Forrest et al. 1990; Yamano and Miwa 1998). Indeed, in neural development, the α and β receptors play distinct roles (Forrest et al. 2002). Studies with *in vitro* cell culture have shown that TRα regulates stem cell proliferation, whereas TRβ mediates differentiation of these newly proliferated cells into neurons (Jones et al. 2003; Lebel et al. 1994; Lezoualc’h et al. 1995).

In the present study, found that dietary PBDE-47 exposure did not affect TR transcripts in the liver, but it decreased mRNA levels for TRβ by 15–22% in the brain of males and females at both doses and elevated TRα transcripts by 37% in females at the high dose. These changes in TR transcript expression may result from the PBDE-induced T_4_ decline or via interactions between PBDE-47 and TRs or their corepressors. It is important to note, however, that transcripts for both TRs are similarly autoinduced by T_3_ in the brain and liver in both sexes in adult fathead minnows (Lema SC, Dickey JT, Schultz IR, Swanson P, unpublished data). That PBDE-47 exposure altered TR transcripts in the brain only, and TRα in a sex-specific pattern, suggests that PBDE-47 or its metabolites act directly on TR gene transcription mechanisms in target tissues (see also Schriks et al. 2007). Moreover, this finding exemplifies how impacts of PBDEs on gene transcripts do not conform to the expectations predicted by general hypothyroidism and demonstrates that these PBDE-induced effects cannot be generalized across tissues or sexes. Although the mechanism for PBDE-47’s impacts on TR gene transcription remains unclear, recent evidence indicates that PBDEs 47, 99, and 209 interact with the mouse pregnane X receptor (PXR) and its human counterpart, the nuclear steroid and xenobiotic receptor (SXR) (Pacyniak et al. 2007). The SXR interacts with the corepressor SMRT (silencing mediator for retinoid and thyroid receptors) (Takeshita et al. 2002), and PBDE-induced impacts on SXR might contribute to changes in TR gene transcription.

Even though the mechanism by which PBDE-47 alters brain TR transcripts is unresolved, our results clearly provide new evidence that dietary intake of PBDEs may impact TH-mediated neural development. In support of this idea, PBDE-47 exposure reduced transcript levels for the TH-regulated gene *BTEB* in the male minnow brain by as much as 53% but did not affect that of females. *BTEB* encodes a zinc-fingered transcription factor that binds GC-box domains to facilitate or inhibit TH-mediated gene transcription. In mammals, T_3_ up-regulation of *BTEB* is specific to neurons (Denver et al. 1999), and in *Xenopus, BTEB* is responsive to T_3_ in brain and other tissues (Furlow and Kanamori 2002; Hoopfer et al. 2002). Studies in which *BTEB* expression has been blocked or induced have revealed that BTEB mediates T_3_-induced neural differentiation and neurite branching via TH activation of *TR*β (Cayrou et al. 2002; Denver et al. 1999). The T_3_-induced BTEB protein also binds the promoter of the *TR*β gene to regulate its autoexpression by THs (Bagamasbad et al. 2008).

Whether the PBDE-induced change in BTEB transcript observed here translates to altered neurogenesis is not clear. Cell proliferation and neural differentiation occur throughout the adult fish brain (Lema et al. 2005; Zupanc et al. 2005), and BTEB transcript is regulated by T_3_ in the brain of adult fathead minnows (Lema SC, Dickey JT, Schultz IR, Swanson P, unpublished data). Still, it is not known if BTEB regulates neurogenesis during this teleost life stage as it does during mammalian development. There is, however, accumulating evidence in mammals that THs regulate adult neurogenesis (Desouza et al. 2005; Fernandez et al. 2004; Tekumalla et al. 2002). In adult rats, for instance, exogenous TH increases immunoreactivity for the cell proliferation marker Ki-67 in the subventricular zone of the brain (Giardino et al. 2000). Taken together, these studies suggest that THs may influence neurogenesis in adults as they do in embryonic and neonatal life.

In summary, our results provide evidence that oral PBDE-47 exposure affects the thyroid axis at several levels by depressing peripheral levels of T_4_, altering pituitary transcripts for TSHβ and GPHα, and changing brain mRNA levels for the TH-responsive genes *TR*α*, TR*β*,* and *BTEB.* Taken together, these results provide evidence that oral intake of the brominated flame retardant PBDE-47 can impact TH-regulated gene transcription in the pituitary gland and brain, and they illustrate how PBDE-induced changes in TH-regulated transcripts do not conform to the effects predicted by general hypothyroidism. Given these findings, it becomes crucial to ask whether these changes in TH-mediated mRNA levels translate into health consequences for humans or wildlife (Birnbaum and Staskal 2004; McDonald 2002). PBDEs are lipophilic and bioaccumulate, and several studies have shown rising PBDE contaminants in the tissues of invertebrates, fish, birds, and marine mammals, as well as in the breast milk, blood, and tissues of humans (Hites 2004; Law et al. 2003; Schecter et al. 2003, 2005). The PBDE-induced changes in TH-regulated gene transcripts seen here indicate that neurogenesis and brain development may be impacted by PBDE exposure, and they highlight the need for future investigations into how PBDEs influence TH-mediated neural function.

## References

[b1-ehp-116-1694] Adolf B, Chapouton P, Lam CS, Topp S, Tannhäuser B, Strähle U (2006). Conserved and acquired features of adult neurogenesis in the zebrafish telencephalon. Dev Biol.

[b2-ehp-116-1694] Ankley GT, Villeneuve DL (2006). The fathead minnow in aquatic toxicology: past, present and future. Aquat Toxicol.

[b3-ehp-116-1694] Bagamasbad P, Howdeshell KL, Sachs LM, Demeneix BA, Denver RJ (2008). A role for basic transcription element-binding protein 1 (BTEB 1) in the autoinduction of thyroid hormone receptor β. J Biol Chem.

[b4-ehp-116-1694] Birnbaum LS, Staskal DF (2004). Brominated flame retardants: cause for concern?. Environ Health Perspect.

[b5-ehp-116-1694] Boas M, Feldt-Rasmussen U, Skakkebæk NE, Main KM (2006). Environmental chemicals and thyroid function. Eur J Endocrinol.

[b6-ehp-116-1694] Cayre M, Malaterre J, Scotto-Lomassese S, Strambi C, Strambi A (2002). The common properties of neurogenesis in the adult brain: from invertebrates to vertebrates. Comp Biochem Physiol B.

[b7-ehp-116-1694] Cayrou C, Denver RJ, Puymirat J (2002). Suppression of the basic transcription element-binding protein in brain neuronal cultures inhibits thyroid hormone-induced neurite branching. Endocrinology.

[b8-ehp-116-1694] Denver RJ, Ouellet L, Furling D, Kobayashi A, Fujii-Kuriyama Y, Puymirat J (1999). Basic transcription element-binding protein (BTEB) is a thyroid hormone-regulated gene in the developing central nervous system. J Biol Chem.

[b9-ehp-116-1694] Desouza LA, Ladiwala U, Daniel SM, Agashe S, Vaidya RA, Vaidya VA (2005). Thyroid hormone regulates hippocampal neurogenesis in the adult rat brain. Mol Cell Neurosci.

[b10-ehp-116-1694] Dickhoff WW, Folmar LC, Mighell JL, Mahnken CVW (1982). Plasma thyroid hormones during smoltification of yearling and underyearling coho salmon and yearling Chinook salmon and steelhead trout. Aquaculture.

[b11-ehp-116-1694] Fernandez M, Pirondi S, Manservigi M, Giardino L, Calzà L (2004). Thyroid hormone participates in the regulation of neural stem cells and oligodendrocyte precursor cells in the central nervous system of adult rat. Eur J Neurosci.

[b12-ehp-116-1694] Fernie KJ, Shutt JL, Mayne G, Hoffman D, Letcher RJ, Drouillard KG (2005). Exposure to polybrominated diphenyl ethers (PBDEs): changes in thyroid, vitamin A, glutathione homeostasis, and oxidative stress in American kestrels (*Falco sparverius*). Toxicol Sci.

[b13-ehp-116-1694] Forrest D, Reh TA, Rüsch A (2002). Neurodevelopmental control by thyroid hormone receptors. Curr Opin Neurobiol.

[b14-ehp-116-1694] Forrest D, Sjöberg M, Vennström B (1990). Contrasting developmental and tissue-specific expression of α and β thyroid hormone receptor genes. EMBO J.

[b15-ehp-116-1694] Furlow JD, Kanamori A (2002). The transcription factor basic transcription element-binding protein 1 is a direct thyroid hormone response gene in the frog *Xenopus laevis*. Endocrinology.

[b16-ehp-116-1694] Giardino L, Bettelli C, Calzà L (2000). In vivo regulation of precursor cells in the subventricular zone of adult rat brain by thyroid hormone and retinoids. Neurosci Lett.

[b17-ehp-116-1694] Gould E, Butcher LL (1989). Developing cholinergic basal fore-brain neurons are sensitive to thyroid hormone. J Neurosci.

[b18-ehp-116-1694] Hallgren S, Sinjari T, Håkansson H, Darnerud PO (2001). Effects of polybrominated diphenyl ethers (PBDEs) and polychlorinated biphenyls (PCBs) on thyroid hormone and vitamin A levels in rats and mice. Arch Toxicol.

[b19-ehp-116-1694] Hamers T, Kamstra JH, Sonneveld E, Murk AJ, Kester MHA, Andersson PL (2006). *In vitro* profiling of the endocrine-disrupting potency of brominated flame retardants. Toxicol Sci.

[b20-ehp-116-1694] Harju M, Hamers T, Kamstra JH, Sonneveld E, Boon JP, Tysklind M (2007). Quantitative structure-activity relationship modeling on in vitro endocrine effects and metabolic stability involving 26 selected brominated flame retardants. Environ Toxicol Chem.

[b21-ehp-116-1694] Hites RA (2004). Polybrominated diphenyl ethers in the environment and in people: a meta-analysis of concentrations. Environ Sci Technol.

[b22-ehp-116-1694] Hoopfer ED, Huang L, Denver RJ (2002). Basic transcription element binding protein is a thyroid hormone-regulated transcription factor expressed during metamorphosis in *Xenopus laevis*. Develop Growth Differ.

[b23-ehp-116-1694] Jones I, Srinivas M, Ng L, Forrest D (2003). The thyroid hormone receptor β gene: structure and functions in the brain and sensory systems. Thyroid.

[b24-ehp-116-1694] Koibuchi N, Chin WW (2000). Thyroid hormone action and brain development. Trends Endocrinol Metabol.

[b25-ehp-116-1694] König S, Neto VM (2002). Thyroid hormone actions on neural cells. Cell Mol Neurobiol.

[b26-ehp-116-1694] Law RJ, Alaee M, Allchin CR, Boon JP, Lebeuf M, Lepom P (2003). Levels and trends of polybrominated diphenyl ethers and other brominated flame retardants in wildlife. Environ Int.

[b27-ehp-116-1694] Lebel JM, Dussault JH, Puymirat J (1994). Overexpression of the beta 1 thyroid receptor induces differentiation in neuro-2a cells. Proc Natl Acad Sci USA.

[b28-ehp-116-1694] Leino RL, Jensen KM, Ankley GT (2005). Gonadal histology and characteristic histopathology associated with endocrine disruption in the adult fathead minnow (*Pimephales promelas*). Environ Toxicol Pharmacol.

[b29-ehp-116-1694] Lema SC, Dickey JT, Swanson P (2008). Molecular cloning and sequence analysis of multiple cDNA variants for thyroid-stimulating hormone β subunit (TSHβ) in the fathead minnow (*Pimephales promelas*). Gen Comp Endocrinol.

[b30-ehp-116-1694] Lema SC, Hodges MJ, Marchetti MP, Nevitt GA (2005). Proliferation zones in the salmon telencephalon and evidence for environmental influence on proliferation rate. Comp Biochem Physiol A.

[b31-ehp-116-1694] Lema SC, Nevitt GA (2004). Evidence that thyroid hormone induces olfactory cellular proliferation in salmon during a sensitive period for imprinting. J Exp Biol.

[b32-ehp-116-1694] Lema SC, Schultz IR, Scholz NL, Incardona JP, Swanson P (2007). Neural defects and cardiac arrhythmia in fish larvae following embryonic exposure to 2,2′,4,4′-tetrabromo-diphenyl ether (PBDE 47). Aquat Toxicol.

[b33-ehp-116-1694] Lezoualc’h F, Seugnet I, Monnier AL, Ghysdael J, Behr J-P, Demeneix BA (1995). Inhibition of neurogenic precursor proliferation by antisense α thyroid hormone receptor oligonucleotides. J Biol Chem.

[b34-ehp-116-1694] Liu Y-W, Lo L-J, Chan W-K (2000). Temporal expression and T3 induction of thyroid hormone receptors α1 and β1 during early embryonic and larval development in zebrafish, *Danio rerio*. Mol Cell Endocrinol.

[b35-ehp-116-1694] Marsh G, Bergman A, Bladh LG, Gillner M, Jakobsson E (1998). Synthesis of Ú-hydroxybromodiphenyl ethers and binding to the thyroid receptor. Organohal Compounds.

[b36-ehp-116-1694] McDonald TA (2002). A perspective on the potential health risks of PBDEs. Chemosphere.

[b37-ehp-116-1694] Meerts IA, van Zanden JJ, Luijks EA, Leeuwen-Bol I, Marsh G, Jakobsson E (2000). Potent competitive interactions of some brominated flame retardants and related compounds with human transthyretin *in vitro*. Toxicol Sci.

[b38-ehp-116-1694] Mörck A, Hakk H, Örn U, Wehler EK (2003). Decabromodiphenyl ether in the rat: absorption, distribution, metabolism, and excretion. Drug Metab Dispos.

[b39-ehp-116-1694] Morgado I, Hamers T, Van der Ven L, Power DM (2007). Disruption of thyroid hormone binding to sea bream recombinant transthyretin by ioxinyl and polybrominated diphenyl ethers. Chemosphere.

[b40-ehp-116-1694] Muirhead EK, Skillman AD, Hook SE, Schultz IR (2006). Oral exposure of PBDE-47 in fish: toxicokinetics and reproductive effects in Japanese medaka (*Oryzias latipes*) and fathead minnows (*Pimephales promelas*). Environ Sci Technol.

[b41-ehp-116-1694] National Center for Biotechnology Information (2008). GenBank Overview.

[b42-ehp-116-1694] Opitz R, Lutz I, Nguyen N-H, Scanlan TS, Kloas W (2006). Analysis of thyroid hormone receptor βA mRNA expression in *Xenopus laevis* tadpoles as a means to detect agonism and antagonism of thyroid hormone action. Toxicol Appl Pharmacol.

[b43-ehp-116-1694] Pacyniak EK, Cheng X, Cunningham ML, Crofton K, Klaassen CD, Guo GL (2007). The flame retardants, polybrominated diphenyl ethers, are pregnane X receptor activators. Toxicol Sci.

[b44-ehp-116-1694] Paternostro MA, Meisami E (1994). Quantitative [^3^H] thymidine autoradiography of neurogenesis in the olfactory epithelium of developing normal, hypothyroid, and hyperthyroid-rehabilitated rats. Dev Brain Res.

[b45-ehp-116-1694] Porterfield SP (2000). Thyroidal dysfunction and environmental chemicals—potential impact on brain development. Environ Health Perspect.

[b46-ehp-116-1694] Porterfield SP, Hendrich CE (1993). The role of thyroid hormones in prenatal and neonatal neurological development–current perspectives. Endocr Rev.

[b47-ehp-116-1694] Schecter A, Päpke O, Tung KC, Joseph J, Harris TR, Dahlgren J (2005). Polybrominated diphenyl ether flame retardants in the U.S. population: current levels, temporal trends, and comparison with dioxins, dibenzofurans, and poly-chlorinated biphenyls. J Occup Environ Med.

[b48-ehp-116-1694] Schecter A, Pavuk M, Päpke O, Ryan KK, Birnbaum L, Rosen R (2003). Polybrominated diphenyl ethers (PBDEs) in U.S. mothers’ milk. Environ Health Perspect.

[b49-ehp-116-1694] Schriks M, Roessig JM, Murk AJ, Furlow JD (2007). Thyroid hormone receptor isoform selectivity of thyroid hormone disrupting compounds quantified with an *in vitro* reporter gene assay. Environ Toxicol Pharmacol.

[b50-ehp-116-1694] Skarman E, Darnerud PO, Ohrvik H, Oskarsson A (2005). Reduced thyroxine levels in mice perinatally exposed to polybrominated diphenyl ethers. Environ Toxicol Pharmacol.

[b51-ehp-116-1694] Takeshita A, Taguchi M, Koibuchi N, Ozawa Y (2002). Putative role of the orphan nuclear receptor SXR (steroid and xenobiotic receptor) in the mechanism of CYP3A4 inhibition by xenobiotics. J Biol Chem.

[b52-ehp-116-1694] Tekumalla PK, Tontonoz M, Hesla MA, Kirn JR (2002). Effects of excess thyroid hormone on cell death, cell proliferation, and new neuron incorporation in the adult zebra finch telencephalon. J Neurobiol.

[b53-ehp-116-1694] Tomy GT, Palace VP, Halldorson T, Braekevelt E, Danell R, Wautier K (2004). Bioaccumulation, biotransformation, and biochemicals effects of brominated diphenyl ethers in juvenile lake trout (*Salvelinus namaycush*). Environ Sci Technol.

[b54-ehp-116-1694] Uchida K, Yonezawa M, Nakamura S, Kobayashi T, Machida T (2005). Impaired neurogenesis in the growth-retarded mouse is reversed by T_3_ treatment. NeuroReport.

[b55-ehp-116-1694] Yamano K, Miwa S (1998). Differential gene expression of thyroid hormone receptor α and β in fish development. Gen Comp Endocrinol.

[b56-ehp-116-1694] Zhou T, Ross DG, DeVito MJ, Crofton KM (2001). Effects of short-term *in vivo* exposure to polybrominated diphenyl ethers on thyroid hormones and hepatic enzyme activities in weanlings rats. Toxicol Sci.

[b57-ehp-116-1694] Zhou T, Taylor MM, DeVito MJ, Crofton KM (2002). Developmental exposure to brominated diphenyl ethers results in thyroid hormone disruption. Toxicol Sci.

[b58-ehp-116-1694] Zupanc GKH, Hinsch K, Gage FH (2005). Proliferation, migration, neuronal differentiation, and long-term survival of new cells in the adult zebrafish brain. J Comp Neurol.

